# Rhein Derivative 4F Inhibits the Malignant Phenotype of Breast Cancer by Downregulating Rac1 Protein

**DOI:** 10.3389/fphar.2020.00754

**Published:** 2020-05-28

**Authors:** Xinxiao Li, Yunfeng Liu, Yuhua Zhao, Wei Tian, Lina Zhai, Huifeng Pang, Jiankang Kang, Huaxin Hou, Yanhua Chen, Danrong Li

**Affiliations:** ^1^Department of Basic Research, Guangxi Medical University Cancer Hospital, Nanning, China; ^2^College of Pharmacy, Guangxi Medical University, Nanning, China; ^3^Life Sciences Institute, Guangxi Medical University, Nanning, China

**Keywords:** Rhein derivative, drug design, Rac family small GTPase 1, breast cancer, anticancer

## Abstract

**Background:**

Triple-negative breast cancer is a common malignant tumor with unfavorable prognosis affecting women worldwide; thus, there is an urgent need for novel therapeutic drugs with improved anti-tumor activity. Rac family small GTPase 1 (Rac1) plays an important role in malignant behavior and is a promising therapeutic target. We reported an anthraquinone compound, Rhein, and its derivative, 4F, and investigated their downregulation effects on Rac1 in breast cancer cells *in vitro*.

**Methods:**

The inhibition of cell proliferation by derivative 4F was investigated in two breast cancer (MDA-MB-231 and MCF-7) and normal breast (MCF-10A) cell lines by cell counting kit-8 assay and growth curves. The role of 4F in cell migration and invasion and cytoskeletal change were assessed by Transwell chamber assay and F-actin staining, respectively. The affinity of Rhein and its derivative for Rac1 protein and the regulation of Rac1 promoter activity were evaluated by molecular docking software and luciferase reporter gene assay, respectively. Rac1 protein expression was determined by western blot assay.

**Results:**

Compared to Rhein, derivative 4F more strongly inhibited breast cancer cell proliferation, migration, and invasion and also cause cytoskeletal changes like those in paclitaxel. Derivative 4F not only bound more stably to Rac1 but also inhibited Rac1 promoter activity in cells and downregulated Rac1 protein expression.

**Conclusions:**

Rhein derivative 4F is a new anthraquinone compound with better anti-tumor activity than that of the lead compound Rhein in breast cancer. It down-regulated Rac1 expression and may be a small molecule inhibitor of Rac1.

## Introduction

Breast cancer is one of the most common malignant tumors in women worldwide. In the past decade, the overall breast cancer incidence rates among Asians/Pacific Islanders increased at an annual rate of 1.7 percent, faster than that of other ethnic groups (DeSantis et al., 2017). Triple-negative breast cancer is characterized by negativity for estrogen receptor (ER), progesterone receptor (PR), and human epidermal growth factor receptor (HER-2). Patients with triple-negative breast cancer typically have unfavorable prognosis owing to a lack of recognized molecular targets for therapy. Chemotherapy is the only established therapeutic option for triple-negative breast cancer, for which anthraquinone anti-tumor antibiotics are the cornerstone (Bianchini et al., 2016; Denkert et al., 2017). However, despite triple-negative breast cancer responses to chemotherapy in the early stage of treatment, drug resistance tends to develop rapidly due to cancer cell's heterogeneity (Cao et al., 2019). Hence, there is an urgent need for novel therapeutic drugs to more efficiently combat triple-negative breast cancer.

**Figure 1:**
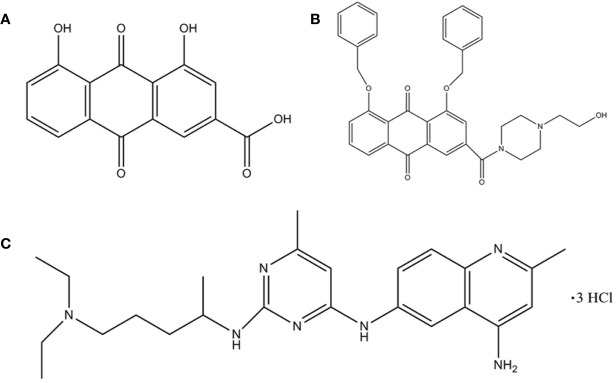
The structure of the ligand molecules drawn in Chem Office Ultra 12.0 software. Both Rhein acid and derivative 4F contain an anthraquinone ring, while derivative 4F and NSC23766 contain nitrogen heterocyclic rings in common. Molecular structure of **(A)** Rhein, **(B)** derivative 4F, and **(C)** NSC23766.

Rhein (1,8-dihydroxy-3-carboxy anthraquinone) (**Figure 1A**) is an anthraquinone compound extracted from plants such as rhubarb and polygonum multiflorum (Lu et al., 2016). Recent studies have shown that Rhein exhibited broad-spectrum anticancer activity by a variety of mechanisms in tongue cancer, stomach cancer, liver cancer, lung adenocarcinoma, granulocyte leukemia, and breast cancer (Shi et al., 2008; Chen et al., 2010; Fernand et al., 2011; Li et al., 2012; Zhou et al., 2015; Wu et al., 2017), suggesting the potential of Rhein as a lead compound in the design of anticancer drugs. However, Rhein is insoluble in water (Yajun and Zhen, 2009), which limits its bioavailability.

Rac family small GTPase 1 (Rac1) is a member of the Rac subfamily of the GTPase Rho family (Payapilly and Malliri, 2018). It is encoded by *RAC1* to produce a variety of alternatively spliced Rac1 proteins that play important roles in cell growth, cytoskeletal reorganization, protein kinase activation, cell cycle, cell adhesion, and cell movement (Bosco et al., 2009). A recent study published in *Nature* suggested that Rac1 may be a priority target for cancer therapy, with evidence to support its feasibility (Behan et al., 2019).

In a previous study, we screened different anthraquinone compounds and a series of tumor proteins with different docking software and scoring functions or algorithms by molecular docking computer-aided drug design and found that Rhein can stably bind Rac1 (Jing et al., 2015; Zhengying et al., 2017). Our previous tumor biological experiments also showed that Rhein plays an anti-tumor role by combining Rac1 (Tang et al., 2016; Zhou et al., 2017). To improve the anti-tumor activity of Rhein, based on previous studies, we modified the structure of Rhein and synthesized a series of derivatives, including derivative RP-4 which enhanced the radiosensitivity of nasopharyngeal carcinoma (Su et al., 2019) and derivative 4a, which inhibited ovarian cancer cell migration and invasion (Pang et al., 2020) and killed liver cancer cells by inducing para-apoptosis (Tian et al., 2019), etc. Among them, derivative 4F showed superior anti-breast cancer effects and good bioavailability. The present study further investigated the inhibitory effect of derivative 4F on breast cancer cell proliferation, invasion, and metastasis *in vitro* and explored its mechanisms, especially the regulation of Rac1 protein expression.

## Materials and Methods

### Reagents

The chemical formula of derivative 4F is C_35_H_33_N_2_O_6_ (1, 8-bis (benzyloxy)-3-(4-(2-hydroxyethyl) piperazine-1-carbonyl)-9,10-anthraquinone) **(****Figure 1B****)**, with a relative molecular mass of 577 and >98% purity [high-performance liquid chromatography (HPLC)]. Derivative 4F is a yellow flaky crystal that is slightly soluble in water and soluble in organic solvents such as dimethyl sulfoxide (DMSO), with a melting point of 155–157°C. Rhein was purchased from Langze (Nanjing, China), vincristine (VCR) was purchased from Wanle (Shenzhen, China), paclitaxel (PTX) was purchased from Yangzijiang (Jiangsu, China), cisplatin (DDP) was purchased from Haosen (Jiangsu, China), and Rac1 activator PMA and Rac1 inhibitor NSC23766 were purchased from Selleck (Houston, USA).

### Cell Culture

The human triple-negative breast cancer MDA-MB-231 and ER-sensitive breast cancer MCF-7 cell lines were obtained from the American Type Culture Collection (ATCC). The human normal breast MCF-10A cell line was obtained from the China Center for Type Culture Collection (CCTCC). Cells were cultured in Dulbecco's Modified Eagle's Medium (DMEM) containing 10% fetal bovine serum and penicillin (100 U/ml) and streptomycin (100 mg/L) in a humidified incubator with a volume fraction of 5% CO_2_ at 37°C.

### Cell Proliferation Assay

Breast cancer MDA-MB-231 cells, MCF-7 cells, and human normal breast MCF-10A cells were used to evaluate cell viability. The cells were incubated in a 96-well plate at a density of 5,000 cells/well for 24 h and then exposed to different concentrations of Rhein (10-320 μmol/L) or derivative 4F (2.5-40 μmol/L). Cells without any drug were used a control. After incubation, 100 μL of 10% cell counting kit-8 (CCK-8) solution was added to each well by liquid exchange method and incubated for 1 h. The absorbance value [optical density (OD)] of each well was measured at 450 nm on a Synergy H1 microplate reader (BioTek Instruments, Inc., USA). The half-maximal inhibitory concentration (IC_50_) was calculated using IBM SPSS Statistics for Windows, version 20.0. after 48 h of treatment. We plotted the growth curves of MDA-MB-231 and MCF-7 cells and calculated the doubling times. Cells were added to each well of a 6-well plate and incubated for 24 h before 4 μmol/L Rhein or derivative 4F were added. One 6-well plate was taken every 24 h to collect cells from each well to count by cytometer (Beckman Culter Z 1, USA). The doubling time (DT) was calculated as follows:

$$\text{DT}=\text{t}\times \frac{\text{lg}2}{\text{lg}{\text{N}}_{\text{t}}-\text{lg}{\text{N}}_{0}}$$

where the *t* was the culture time, *N_o_* was the number of cells recorded for the first time (24 h after cell inoculation), and *N_t_* was the number of cells after time *t*.

### Cell Migration and Invasion Assay

After withdrawing the fetal bovine serum, the cells were starved for 24 h. A total of 4×10^4^ cells (200 μl) were added to the Transwell chamber and 600 μl of a medium containing 20% fetal bovine serum was added to the lower chamber of the 24-well plate. After adding 100 μl of Rhein or derivative 4F to the chamber, the final drug concentrations were 8 μmol/L. The control was added with 100 μl serum-free medium. The cells were incubated for 24 h; next, the cells and liquid on the surface of the upper chamber membrane were wiped with a cotton swab, fixed with methanol, air-dried with phosphate-buffered saline (PBS), and stained with 0.2% crystal violet. Each chamber was photographed under a microscope at 20× magnification from five different fields of view: upper, lower, left, right, and middle. The migrated cells were finally counted using ImageJ. Cell invasion ability was measured similarly except that the bottom of the Transwell chamber membrane was coated with Matrigel before the cells were added.

### Cytoskeleton Staining Assay

A total of 2×10^4^ cells were incubated in each well of a 6-well plate with a built-in sterile cell slide for 24 h and then treated with drugs (Rhein 50 μmol/L, derivative 4F 8 μmol/L, VCR 200 nmol/L, PTX 100 nmol/L, DDP 10 μmol/L) for 48 h. The cell slides were washed with PBS, fixed with ice-cold acetone, and blocked with 0.2% TritonX-100 and 1% bovine serum albumin (BSA). The samples were placed in an icebox and F-actin reagent R37110 (Life Technologies, USA) and PBS were added and the samples were slowly shaken for 30–45 min. Next, the dye solution was removed and 4',6-diamidino-2-phenylindole (DAPI) was added to dye the nucleus. The samples were then washed with PBS, blocked with glycerin, and the cytoskeleton observed and photographed by laser confocal microscopy (Nikon A1, Japan) from 3 different fields of view. The assay was repeated for 3 times.

### Molecular Docking

Molecular Operating Environment (MOE. 2008, CCG Montreal, Canada) (Vilar et al., 2008) was used to study the interaction between compound molecules and Rac1 protein as a receptor. The ASE scoring function in MOE was used to calculate the conformational strength of each compound bound to Rac1, with the lowest ASE score indicating the optimal conformation. The ligand molecules were Rhein, derivative 4F, and the positive control NSC23766 **(****Figure 1C****)**.

### Luciferase Reporter Assay

A lentivirus containing a RAC1-promoter-Luc2 plasmid was previously developed by our group and verified by double enzyme digestion assay (Li et al., 2019). Human RAC1 promoter (GenBank: NM_006908) was used as the target sequence to design and synthesize the pUC57-RAC1-promoter-Luc2 vector and recombined with the pLVX-AcGFP-Puro vector to construct the pLVX-RAC1-Luc2-GFP-Puro plasmid so that the RAC1 gene promoter was accurately inserted upstream of the luciferase gene. Lipofectamine™2000 reagent was then used to transfect the recombinant and viral package plasmids into 293T cells for packaging. The lentiviral supernatant containing RAC1-promoter-Luc2 plasmid was collected for future use. In this study, MDA-MB-231 cells at a density of 5×10^4^ cells/ml were seeded into 6-well plates and 500 μl of virus solution was added 24 h later. After 48 h of continuous infection, the cells were seeded into a T25cm^2^ culture flask at a dilution of 1:100 so that the cells were completely dispersed and cultured as single cells. After screening for 2 weeks with medium containing puromycin (2 μg/ml), a cell line stably expressing luciferase, MDA-MB-231-RAC1-Luc2, was obtained. The MDA-MB-231-RAC1-Luc2 cells were incubated in 96-well plates at 1×10^4^ cells/well. After 24 h of adherence, the corresponding drug concentrations (PMA 10 μmol/L, NSC23766 25 μmol/L, Rhein 50 μmol/L, derivative 4F 2, 4, and 8 μmol/L) or serum-free medium were added. The effects of the compounds on luciferase assay in the MDA-MB-231-RAC1-Luc2 cell line were evaluated using the ONE-Glo™ Reagent (Promega, Madison, WI, USA).

### Western Blot Assay

Cells were first treated with different drugs and then collected and lysed with radioimmunoprecipitation assay (RIPA) lysate. After centrifugation, the supernatant was taken. The total protein concentration was determined by bicinchoninic acid (BCA) method and protein samples were denatured at 100°C for 5 min. After electrophoresis with 10% sodium dodecyl sulfonate-polyacrylamide (SDS-PAGE) gel, the proteins were transferred to a 0.22 μm nitrocellulose (NC) membrane. The membrane was blocked with 5% skim milk powder at room temperature for 2 h and incubated with the primary antibodies against glyceraldehyde 3-phosphate dehydrogenase (GAPDH, 1:1000) and Rac1 (1:500) at 4°C overnight. After rinsing with tris-buffered saline, 0.1% Tween 20 (TBST), fluorescence secondary antibody (1:500, 1:10,000) was added and incubated at room temperature for 1.5 h. The proteins bands were quantified using an Odyssey Imaging System (LI-COR, USA).

### Statistics

The experimental results and data were analyzed in SPSS. The measurement data were presented as means ± standard deviation (mean ± SD) and the experiment was repeated three times independently. One-way analysis of variance (ANOVA) was used to compare multiple groups. Least significant difference (LSD) *t*-tests were used for comparisons between groups. *P*<0.05 was considered statistically significant.

## Results

### Derivative 4F Inhibits Breast Cancer Cell Proliferation

**Figure 2:**
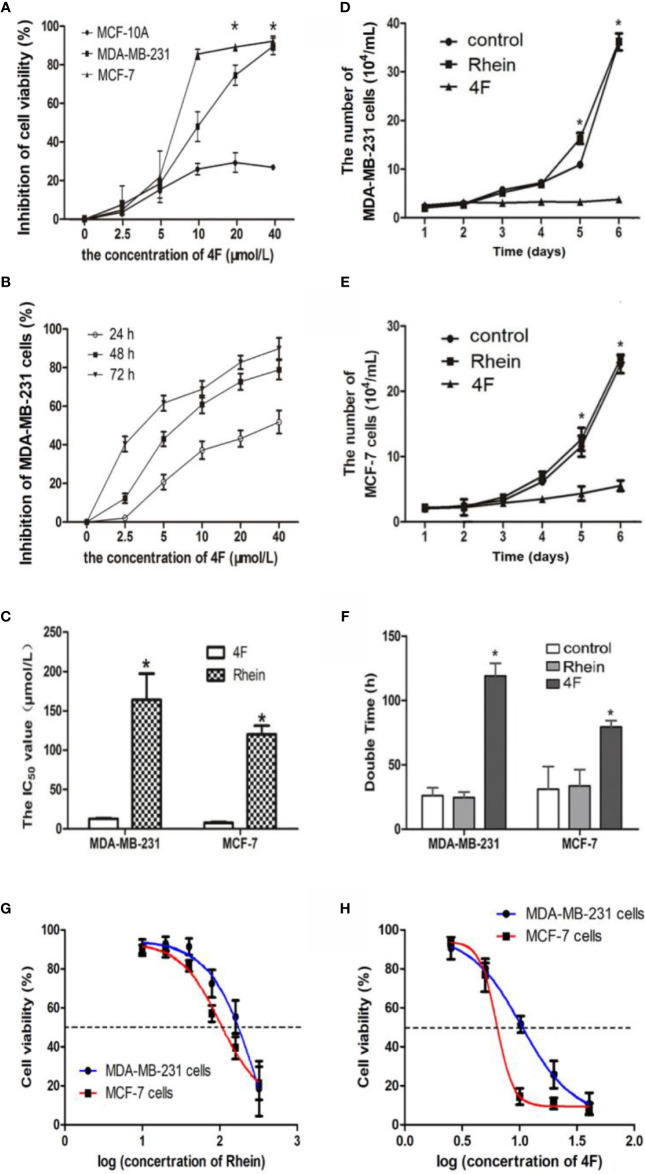
Derivative 4F inhibition of breast cancer or normal cell proliferation. Control group corresponds to cells growing without any drug. Cell numbers were measured by cytometer. IC_50_ values were calculated after 48 h of treatment. Data are shown as means ± SD (n = 3), one-way analysis of variance (ANOVA) was used for statistical analysis. **(A)** Comparison of derivative 4F cytotoxicity to breast cancer and normal breast cells. *P < 0.05, comparison of MCF-10A group and MDA-MB-231 or MCF-7 group. **(B)** MDA-MB-231 cells treated with different concentrations of derivative 4F for 24, 48, and 72 h. **(C)** IC_50_ values of the two breast cancer cell lines. *P < 0.05, comparison of derivative 4F group and Rhein group. **(D)** Growth curve of MDA-MB-231 cells. *P < 0.05, comparison of derivative 4F group and control group. **(E)** Growth curve of MCF-7 cells. *P < 0.05, comparison of derivative 4F group and control group. **(F)** Doubling time of two breast cancer cell lines. *P < 0.05, comparison of derivative 4F group and control group. **(G)** IC_50_ curve of Rhein in MDA-MB-231 and MCF-7 cells. **(H)** IC_50_ curve of derivative 4F in MDA-MB-231 and MCF-7 cells.

Under the same concentrations 2.5-40 μmol/L of derivative 4F treatment, comparison of derivative 4F on the cytotoxicity of breast cancer MDA-MB-231, MCF-7, and normal breast MCF-10A cells, showed significantly lower cytotoxicity of derivative 4F to normal breast cells than that to breast cancer cells (**Figure 2A**). Moreover, its toxicity to human triple-negative breast cancer MDA-MB-231 cells in this concentration range was time- and dose-dependent (**Figure 2B**). The IC_50_ values of derivative 4F and Rhein were (12.80 ± 0.83) and (163.96 ± 33.36) μmol/L in MDA-MB-231 cells and (7.54 ± 1.25) μmol/L and (120.19 ± 10.98) μmol/L in MCF-7 cells (**Figur2C, G, H****) (**Yunfeng et al., 2019a; Yunfeng et al., 2019b). The IC_50_ of derivative 4F was much lower than that of the lead compound Rhein. The growth curves of MDA-MB-231 and MCF-7 cells are shown in **Figures 2D, E**. In both cell lines, after treated with 4 μmol/L Rhein or derivative 4F, cell growth under derivative 4F treatment was much slower than those in the Rhein and control groups. After derivative 4F treatment, the doubling times of MDA-MB-231 and MCF-7 cells were 119.72 and 79.4 h respectively, much longer than the 26.6 and 31.8 h in the control group and 24.5 and 33.5 h in the Rhein group (**Figure 2F**).

### Derivative 4F Inhibits Breast Cancer Cell Migration and Invasion

**Figure 3:**
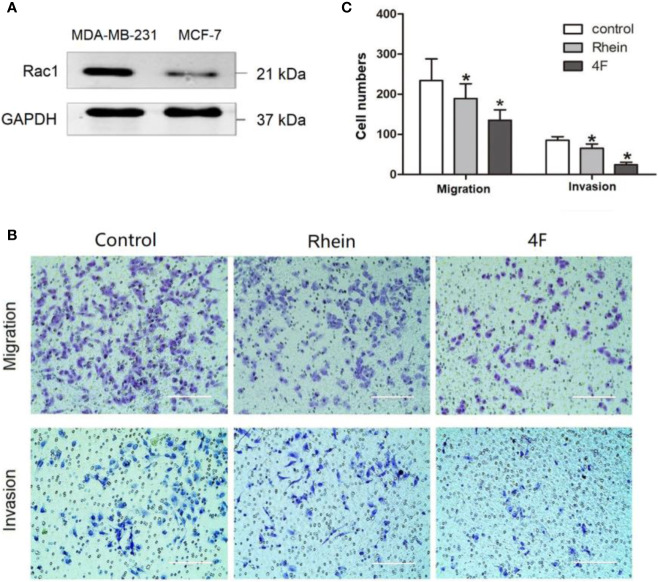
Transwell assay to test MDA-MB-231 cell migration and invasion ability. The control group correspond to cells growing without any drug. **(A)** Rac1 protein expression in MDA-MB-231 and MCF-7 cells. **(B)** Transwell assay of MDA-MB-231 cells (scale bar: 200 μm). **(C)** Numbers migrated and invaded MDA-MB-231 cells. Data are shown as means ± SD (n = 5). One-way analysis of variance (ANOVA) was used for statistical analysis. *P < 0.05, comparison of Rhein group or derivative 4F group and control group on MDA-MB-231 cells.

The migration and invasion ability of the human triple-negative breast cancer MDA-MB-231 cells was significantly higher than that of MCF-7 cells. The western blot revealed higher Rac1 expression in MDA-MB-231 cells than that in MCF-7 cells (**Figure 3A**). Therefore, the MDA-MB-231 cells were used to verify the effect of derivative 4F on cell migration and invasion. The Transwell assay results showed migration cell numbers in the control, Rhein, and derivative 4F groups of (234 ± 54), (189 ± 37), and (135 ± 26), respectively. The numbers of invasive cells were (85 ± 9), (65 ± 10), and (24 ± 6), respectively (**Figures 3B, C**). The numbers in the derivative 4F group were the lowest in both experiments.

### Derivative 4F Affects the Cytoskeleton in Breast Cancer Cells

**Figure 4:**
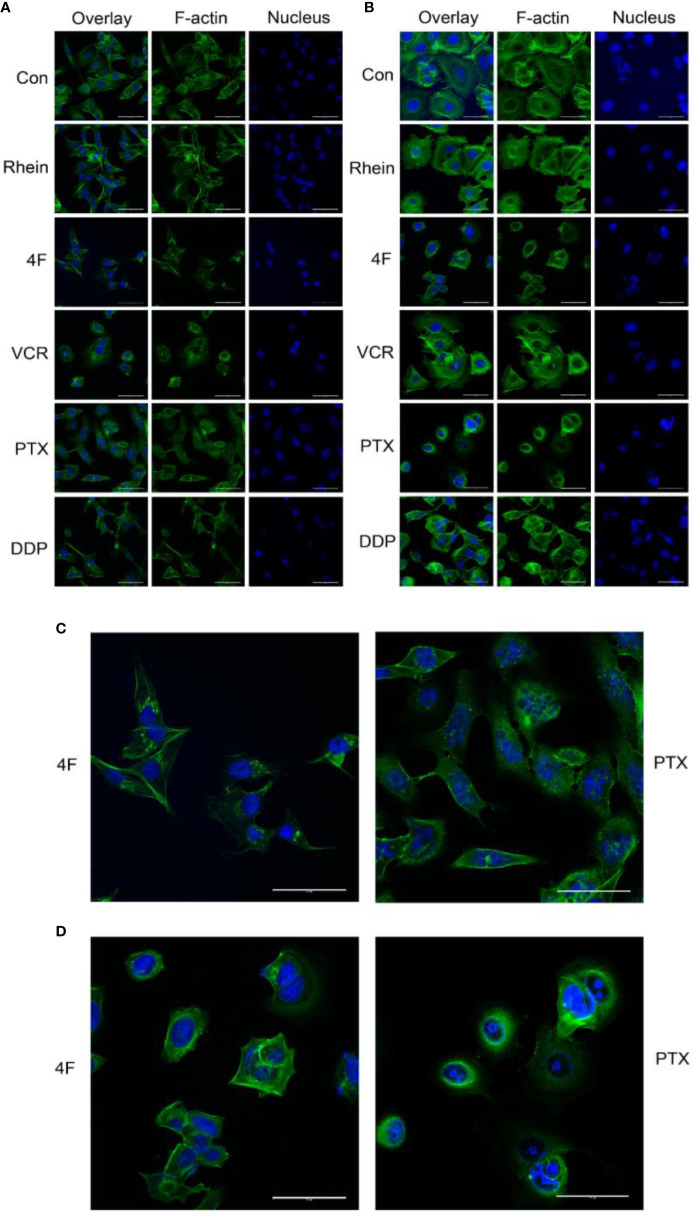
Laser confocal microscopy to detect the effects of different drugs on the cytoskeleton of breast cancer cells (scale bar: 50 μm). F-actin and the nucleus appear in green and blue, respectively. **(A)** Cytoskeletal changes after drug treatments in MDA-MB-231 cells. **(B)** Cytoskeletal changes after drug treatments in MCF-7 cells. **(C)** MDA-MB-231 cells treated with derivative 4F and PTX (scale bar: 50 μm). **(D)** MCF-7 cells treated with derivative 4F and PTX (scale bar: 50 μm).

F-actin staining was used to observe the effects of Rhein and derivative 4F on cellular cytoskeletons, using VCR, PTX, and DDP as positive controls. The main effects of derivative 4F and other drugs on breast cancer MDA-MB-231 and MCF-7 cells were as follows (**Figures 4A, B**): the cells became round and wrinkled, the pseudopodia on the cell membrane disappeared, the intracellular microfilaments were significantly reduced and broken and concentrated around the nucleus, and some elongated microfilaments were disorganized and gathered in the nuclear membrane. Both of them showed similar reactions to those of PTX treatment (**Figures 4C, D**), which induces cell microfilament polymerization and inhibits microfilament depolymerization.

### Molecular Docking of Rac1 Protein and Derivative 4F

**Figure 5:**
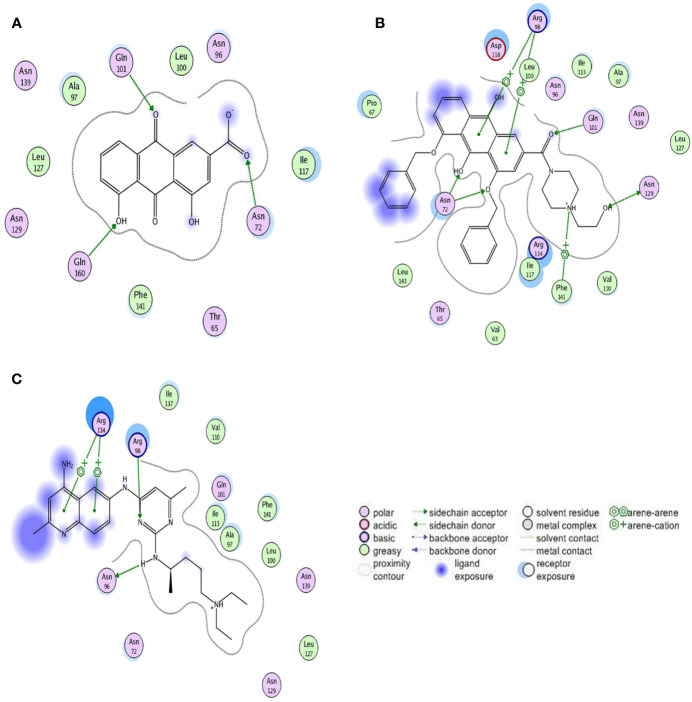
Two-dimensional view of the interaction modes of minimum energy conformations of compounds with Rac1. Hydrogen bonds are shown as dotted arrows, greasy amino acid residues are shown as green circles, and polar amino acid residues are shown as pink circles. **(A)** Rac1-Rhein complex. **(B)** Rac1-derivative 4F complex. **(C)** Rac1-NSC23766 complex.

Three compounds docked with Rac1 respectively. The docking results indicated that the compounds were bound to Rac1 with different conformations. According to the MOE. 2008 docking result, the binding stability of these three compounds to Rac1 is ranked from high to low: derivative 4F> NSC23766> Rhein (**Table 1**), and the optimal binding mode of these compounds with Rac1 protein calculated by ASE scoring function are shown in **Figure 5**.

**Table 1:** ASE function score and molecular force of compounds docked with Rac1.

Compounds	The lowest Binding energy (Kcal)	Hydrogen bond	Distance of hydrogen bond (Å)	Arene-cation interactions	Hydrophobic interactions	
Rhein	-14.4757	Asn 72	2.79		Phe 141	IIe 117
		Gln 160	2.82		Leu 127	
		Gln 101	2.85		Ala 97	Leu 100
Derivative 4F	-22.9834	Asn 129	2.76	Arg 98	Pro 67	IIe 117
		Asn 72	2.66	Arg 98	Leu 143	IIe 113
		Asn 72	2.76	Phe 141	Val 63	Phe 141
		Gln 101	2.57		Val 110	Ala 97
		Asn 129	3.10		Leu 127	Leu 100
NSC23766	-16.7129	Arg 98	2.99	Arg 114	Val 110	Ala 97
		Asn 96	2.05	Arg 114	IIe 117	IIe 113
					Phe 141	Leu 100
					Leu 127	

The ASE scoring function value indicates the stability of the complex, such that a lower score represents a more stable complex. The ASE score mainly depends on the number and distance of hydrogen bonds; however, the number of aromatic cations and interacting amino acid residues also contribute to the complex stability. The binding energies of Rhein, derivative 4F, and NSC23766 docked with Rac1 protein were all less than -7.5 kcal/mol, indicating effective docking (Nicola et al., 2009). Rhein, derivative 4F, and NSC23766 formed three, five, and two hydrogen bonds with Rac1, respectively, indicating the strongest interaction force for derivative 4F. In addition, compared to Rhein and NSC23766, derivative 4F formed the most aromatic cations and amino acid residues with Rac1, which contributed to the stronger hydrophobic and van der Waals force interactions.

### Derivative 4F Inhibits Rac1 Promoter Activity

**Figure 6:**
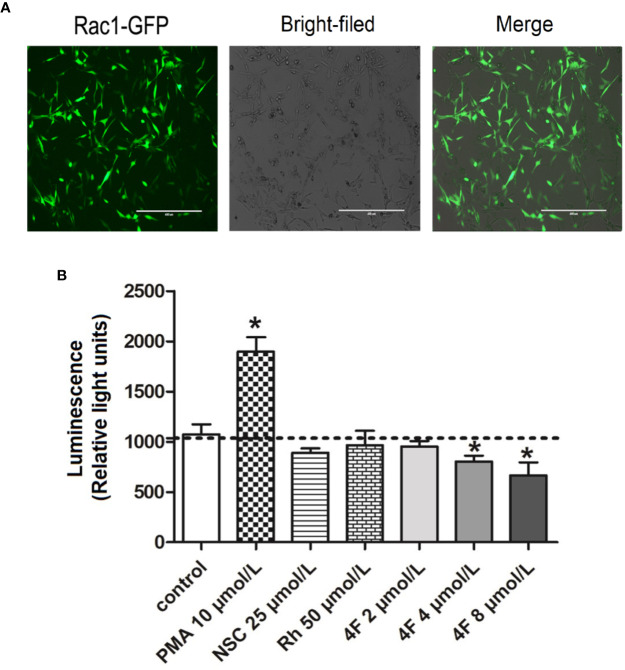
Luciferase activity of Rac1 promoter in MDA-MB-231 cells treated with different compounds. **(A)** GFP-labeled MDA-MB-231 cells following Rac1-promoter-Luc2 lentivirus transfection (scale bar: 400 μm). Under an inverted fluorescence microscope, almost all cells show green fluorescence with moderate intensity with a fluorescent cell count indicating a 90% transfection efficiency. **(B)** Luciferase activity of Rac1. Data are shown as means ± SD (n = 3). One-way analysis of variance (ANOVA) was used for statistical analysis. *P < 0.05, comparison of each compound group and control group.

Luciferase activity reflects the transcriptional activity of the promoter; thus, detecting luciferase expression provides a rapid and cheap method for monitoring promoter activity (Wet et al., 1987). To validate the derivative 4F regulation of Rac1, we constructed an MDA-MB-231-RAC1-Luc2 stable cell line containing the Rac1 promoter in the luciferase reporter vector. After puromycin screening, the transfection efficiency reached 90% (**Figure 6A**), which means the MDA-MB-231-RAC1-Luc2 cells can be used to detect the regulatory effect of different compounds on Rac1 promoter activity. As shown in **Figure 6B**, after the cells were treated with different compounds, Rac1 activator PMA up-regulated the luciferase activity of Rac1, while NSC23766, Rhein, and derivative 4F inhibited Rac1 luciferase activity in cells. The luciferase activities of cells treated with derivative 4F at 4 and 8 μmol/L were significantly reduced, comparable to the decrease observed for NSC23766 but at lower concentrations.

### Derivative 4F Downregulates the Expression of Rac1 Protein

**Figure 7:**
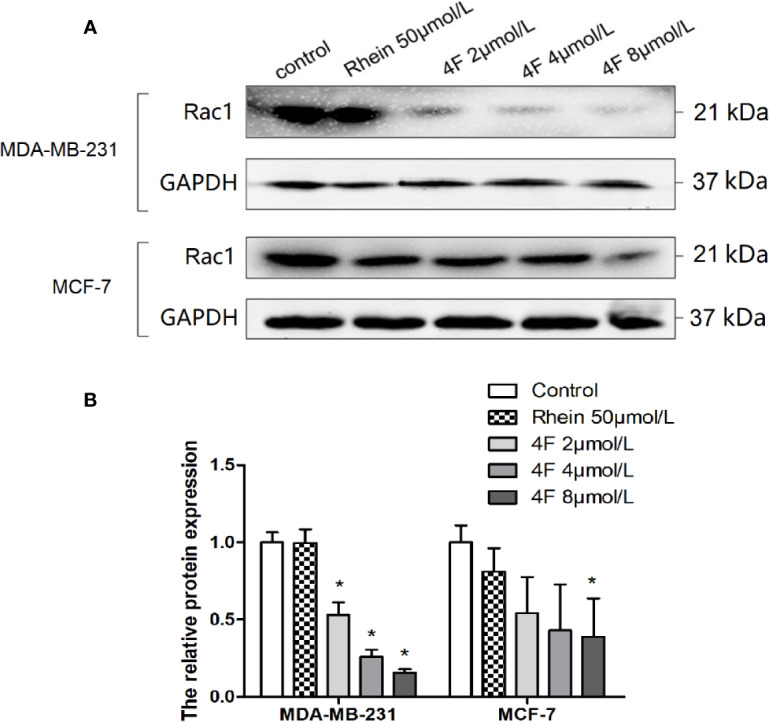
Rac1 protein expression inhibition by Rhein and derivative 4F in breast cancer cells. **(A)** Western blot assay to detect Rac1 protein expression levels in MDA-MB-231 and MCF-7 cells. **(B)** The relative Rac1 protein expressions in MDA-MB-231 and MCF-7 cells. Rac1 expression (normalized against GAPDH) in the control groups was considered to be 100% in these two cell lines. Data are shown as means ± SD (n = 3). One-way analysis of variance (ANOVA) was used for statistical analysis. *P < 0.05, comparison of each treated group and control group.

We used western blot assay to detect Rac1 protein expression in MDA-MB-231 and MCF-7 cells. We found that both Rhein and derivative 4F down-regulated expression in both cell lines, but derivative 4F worked at a lower concentration than did Rhein and had a more obvious effect on human triple-negative breast cancer MDA-MB-231 cells with high Rac1 expression. 2 μmol/L derivative 4F could significantly affect MDA-MB-231 cells, while the corresponding concentration was 8 μmol/L on MCF-7 cells. Moreover, with the increase of derivative 4F concentration, the expression of Rac1 protein decreased in a concentration-dependent manner in both cells (**Figure 7**).

## Discussion

According to the *2019 CSCO Breast Cancer Treatment and Treatment Guide*, chemotherapy is an imperative treatment for triple-negative breast cancer due to the lack of special therapeutic targets. Anthraquinones, taxanes, and platinum-based drugs are commonly used for the treatment of breast cancer. Rhein is a natural anthraquinone compound with low toxicity that is well tolerated by humans. To produce additional anthraquinone compounds, researchers have focused on the structural modification of the 1,8-phenolic hydroxyl groups and 3-carboxy of Rhein as a lead compound (Yao et al., 2014; Jiang et al., 2019). We produced the novel Rhein derivative 4F by introducing a benzyloxy group to the 1,8-phenolic hydroxyl group, which induced endoplasmic reticulum stress, as well as a hydroxyethyl piperazine group to the 3-carboxyl group. Among nitrogen heterocycles, the piperazine motif has attracted considerable attention as its double nitrogen atom and hydrogen in water can form a hydrogen bond effect; thus, it has good hydrophilicity and can significantly increase the water solubility of the drug, while also improving the biological activity and enhancing the action of the drug (Patel and Se Won, 2013; Rathi et al., 2016). We found that derivative 4F inhibited cell growth in a time- and dose-dependent manner and also prevented excessive toxicity in normal breast cells when killing breast cancer cells. Furthermore, its inhibition on the proliferation and growth of breast cancer cells was also much stronger than that of Rhein and control, and greatly prolonging the doubling time of cancer cells. Our previous study also proved that derivative 4F is far less cytotoxic to normal breast cells than doxorubicin (Yunfeng et al., 2019b). This indicates that the side chain of Rhein was successfully modified and that derivative 4F is may be a selective anthracycline candidate with low toxicity. Based on this, we focused on the effect of derivative 4F on the cancer cells in subsequent experiments.

Rac1 is a small (21 kDa) signal GTPase. Numerous studies have suggested that Rac1 may be a therapeutic target for tumors (Ma et al., 2009; McAllister, 2012; Stallings-Mann et al., 2012). Like other members of the Rho subfamily, Rac1 regulates a variety of signaling pathways that control corresponding physiological processes. We used the molecular docking computer-assisted drug design MOE software to evaluate the binding stability of derivative 4F and Rac1. The results showed that derivative 4F formed more hydrogen bonds with Rac1; thus, the interaction force was stronger. In addition, derivative 4F also formed more arene-cation with Rac1 and formed hydrophobic interactions with more amino acid residues than those for Rhein and the positive control Rac1 inhibitor NSC23766. Therefore, the energy of the Rac1-derivative 4F complex was lower and derivative 4F was more stable in combination with Rac1. However, the evaluation of molecular docking software on the effect of compounds and Rac1 protein is only a theoretical speculation, and how the derivative 4F fits in the binding pocket needs to be further confirmed. To rise from theory to practice, we also performed a luciferase reporter gene assay to explore whether derivative 4F had a real regulatory effect on Rac1 in cells. In biological research, luciferase is often used as a reporter gene to evaluate the transcriptional activity of cells transfected with the luciferase gene under the control of the promoters of interest. Our results showed that derivative 4F reduced the luciferase activity of Rac1 in a dose-dependent manner, which means derivative 4F can practically inhibit Rac1 promoter activity in breast cancer cells. Meanwhile, the expression of Rac1 protein was also down-regulated by derivative 4F in a dose-dependent manner, which can prove the effective regulation of derivative 4F on Rac1 to some extent.

Tumor recurrence and metastasis are inseparable from the microscopic infiltration and invasion ability of cells. Cancer cell growth and invasion of local and distant tissues require dysregulation of cell movement, which is an important sign of cancer cell invasion and metastasis. Our study showed higher Rac1 expression in triple-negative breast cancer MDA-MB-231 cells than that in MCF-7 cells, which may explain why MDA-MB-231 cells showed stronger migration and invasion abilities. We also found that both Rhein and derivative 4F inhibited MDA-MB-231 cell migration and invasion, with significantly stronger inhibition by derivative 4F. Thus, derivative 4F, which is structurally modified, was superior to Rhein in inhibiting breast cancer cell migration and invasion. What counts is, this is probably done by affecting Rac1 because the western blot assay showed that derivative 4F could down-regulate Rac1 protein expression in breast cancer cells at a lower effective concentration than that for Rhein. Furthermore, compared to MCF-7 cells, derivative 4F showed a more obvious effect on human triple-negative breast cancer MDA-MB-231 cells with high Rac1 expression. These findings indicate that Rac1 may be an action site of derivative 4F and Rhein and confirm the regulation of Rac1 expression by derivative 4F.

Tumor metastasis requires cytoskeletal remodeling. The cytoskeleton consists of microtubules, microfilaments (actin filaments), and intermediate filaments. It is a complex fibrous network structure. Some classical signaling pathways between microfilaments and their interacting proteins, such as the Rho GTP kinase signaling pathway, are important for tumor metastasis. The Rac family (Rac1, Rac2 and/or Rac3 depending on cell type and condition) can promote the formation of numerous membrane extensions in lamellipodia by mediating actin polymerization and the lamellipodia have been shown to drive movement in many cells *in vivo (*Ridley, 2015). Similarly, Rac1 and other Rho proteins can control actin to affect cytoskeleton and microtubule dynamics through direct interactions with multiple effector proteins (Bosco et al., 2009). In this study, we chose three drugs as positive controls: the anti-mitotic drug VCR, which inhibits tubulin polymerization, and affects spindle microtubule formation; PTX, which induces microfilament polymerization and inhibits cell mitosis; and the cytotoxic drug DDP, which affects cellular DNA. Laser confocal microscopy showed that the effect of derivative 4F on the cytoskeleton was similar to that of PTX, which can induce microfilament polymerization and inhibit microfilament depolymerization. Microtubules and microfilaments are interdependent and highly motile and participate in eukaryotic cell movement (Rosenblum and Shivers, 2000). PTX has been shown to have specific binding sites on microtubules, which can specifically and reversibly bind microtubules and inhibit motility (Parness and Horwitz, 1981; Yang and Horwitz, 2017). These findings suggest that derivative 4F may restrain cell movement by controlling microtubule and microfilament activity, comparable to the action of PTX.

In summary, derivative 4F, a novel Rhein derivative with good cell selectivity, showed stronger lethality to breast cancer cells while being less toxic to normal breast cells. It not only showed a strong ability to bind Rac1 but also reduced the transcriptional activity of the Rac1 promoter, downregulated Rac1 protein expression in cells and caused microfilament rearrangement to inhibit cell invasion and metastasis. These findings suggest that derivative 4F may be a small molecular inhibitor of Rac1 in breast cancer cells. The results of our study provide theoretical support for the structural modification and application development of Rhein and offer new ideas for the design of anti-tumor drugs and the development of therapeutic drugs for triple-negative breast cancer. However, the detailed molecular mechanism and signaling pathway by which derivative 4F regulated Rac1 and its antitumor effects *in vivo* require further study.

## Data Availability Statement

The data supporting the conclusions of this article are available on request to the corresponding authors.

## Ethics Statement

The study was approved by the Ethics Committee of Guangxi Medical University.

## Author Contributions

YL performed and analyzed all the experiments. HH and WT drafted the work for important intellectual content. XL and DL edited the language and figures. YZ, LZ, HP, and JK collected and analyzed the data. YC and HH provided reagents and advice. All authors contributed to manuscript revision, read and approved the submitted version.

## Funding

This work was supported by the National Natural Science Foundation of China (Grant No. 81460561 and 81360502), Guangxi Natural Science Foundation (Grant No. 2018GXNSFAA281064), Innovation Project of Guangxi Graduate Education (Grant No.YCSW2019107), and the Program of Key Laboratory of High-Incidence-Tumor Prevention and Treatment, Guangxi Medical University, the Ministry of Education, China (Grant No. GKE2019-23).

## Conflict of Interest

The authors declare that the research was conducted in the absence of any commercial or financial relationships that could be construed as a potential conflict of interest.
