# Supplementary figures and images for: Rhein Derivative 4F Inhibits the Malignant Phenotype of Breast Cancer by Downregulating Rac1 Protein
Source: Front Pharmacol. 2020 May 28;11:754. doi: 10.3389/fphar.2020.00754 (PMC7274043; doi:10.3389/fphar.2020.00754)

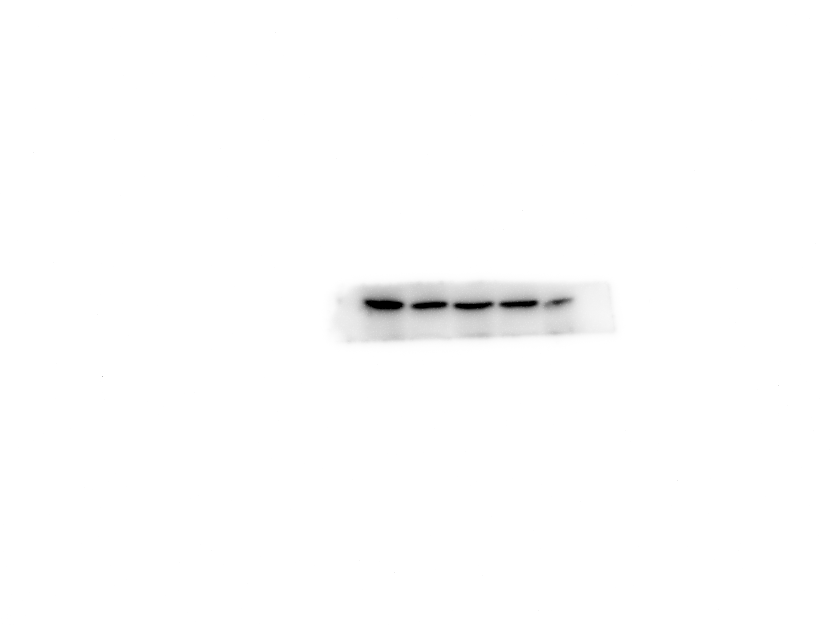

Supplement: Supplementary file 1 [file DataSheet_1.zip › 528190 images/MCF-7 Rac1.tif]

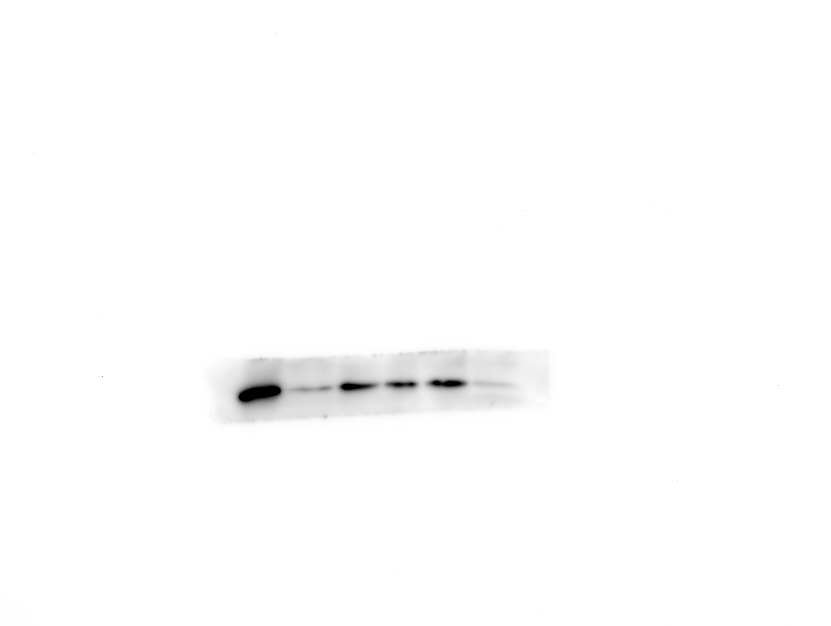

Supplement: Supplementary file 1 [file DataSheet_1.zip › 528190 images/MDA-MB-231 Rac1.tif]
